# Letting stories breathe: Identifying adverse and benevolent childhood experiences in the stories of Military-Connected Children and Young People

**DOI:** 10.1371/journal.pgph.0005471

**Published:** 2025-12-03

**Authors:** Paul G. Watson, Claire Camara, Rachel Cairns, Stephanie Bramwell, Melissa Soto, Elizabeth Crouch

**Affiliations:** 1 Faculty of Health and Life Sciences, Northumbria University, Newcastle upon Tyne, United Kingdom; 2 Public Health Studies, Johns Hopkins University, Baltimore, Maryland, United States of America; 3 Rural and Minority Health Research Centre, Arnold School of Public Health, University of South Carolina, Columbia, South Carolina, United States of America; PLOS: Public Library of Science, UNITED STATES OF AMERICA

## Abstract

This study explores the narrated lived experiences of Military-Connected Children and Young People (MCCYP) in Denmark and examines the relationship between Adverse Childhood Experiences (ACEs) and Benevolent Childhood Experiences (BCEs), particularly in the context of parental combat-related PTSD within their told stories. Using content analysis, interview data was re-analysed using the Adverse Childhood Experiences (ACEs) and Benevolent Childhood Experiences (BCEs) questionnaires to identify ACEs and BCEs within the captured narrative data. The initial study where the data was captured examined military children’s experiences and the impact of a five-day residential camp on well-being, resilience, and self-esteem, based on co-constructed meaning between participants and researchers, with ethical approval ensuring parental consent and participant assent. Ten young people (aged 12–19, mean = 15.00, SD = 2.54) attended the Denmark-based camp run by Støt Soldater & Pårørende (SSOP), a charity supporting children of veterans. Six were female, four males, and all had at least one parent with a self-reported PTSD diagnosis. The findings show that most participants (nine out of ten) had a parent with PTSD, leading to an average ACE score of 2.7. These challenges included physical or emotional abuse, living with a parents who has poor mental health, witnessing domestic violence and having a parent abuse substances. Despite these challenges, all participants reported key protective factors, contributing to an average BCEs score of 4. The protective factors included feeling safe with a caregiver, having external support, and experiencing home stability. The study discusses the implications for clinical practice, proposing the ICE (Identify, Connect, Engage) model for Trauma-Informed Care (TIC), which focuses on early identification of adversities, building trust through compassionate connection, and involving MCCYP in decision-making. The study underscores the importance of letting stories breathe to considering both the adversities and resilience factors in MCCYP narratives, advocating for a holistic, child-centred approach to supporting their health and well-being.

## Introduction/Background

Military connected children and young people (MCCYP) face numerous challenges and stressors which include the impact of parental deployment, parental illness or injury, bereavement, family relationship breakdown. These adversities can have a cumulative impact on a MCCYP physical and emotional well-being, documented as Adverse Childhood Experiences (ACEs) which can lead to long term consequences in adulthood [1,2]. The harmful experiences, known as adverse childhood experiences (ACEs), consist of physical abuse and/ or emotional abuse, sexual abuse, physical and/or emotional neglect, substance abuse within the household, violent behaviour towards the mother, parental separation or divorce, mental illness in the household, and having a family member incarcerated, were initially identified by Felitti, Schestatsky, Knijnik et al [3]. The ACEs framework demonstrates the connection between childhood exposure to adverse experiences and poor health outcomes [3]. These negative adverse experiences can take place at any point in life, often varying in intensity and severity [4].

Interest in a particular subgroup, specifically children from military families, has intensified due to research indicating that they may be more susceptible to ACEs due to the unique challenges associated with military life [5–8]. Whilst a large proportion of research on children in military families focuses on the challenges, Crouch, Anderson, and Smith [7] highlight an incomplete picture surrounding the strengths and resilience built among military children who may endure adverse experiences. As we continue to develop our understanding and awareness of the impact and prevalence of ACEs in military families, it is imperative that we also focus on uncovering these experiences and building on the support mechanisms and strategies for military children and young people.

According to Ministry of Defence (MOD) data presented in [2] 79% of UK service personnel have children. This is an increase of 25% since 2009, and totals between 91000 and 115000 children and young people [2]. While the difficulties faced by Military-Connected Children and Young People (MCCYP) have been researched and outlined [9,10], there has been little to no evidence to understand the positive aspects of military life. Examining positive childhood experiences, otherwise known as benevolent childhood experiences (BCEs), alongside ACEs is imperative to understanding both the direct effect of BCEs on long-term health outcomes and the impact of BCEs on the effects of ACEs [11]. Although there is evidence that BCEs may play a positive role independently and a protective role when examined with ACEs [12,13], there is limited research examining the co-occurrence and interplay of ACEs and BCEs within the population of MCCYP.

### The study

The research reported in this paper is part of the narrated data collection from a previous project involving an evaluation of Danish MCCYP emotional health and well-being during their attendance to a summer camp provided by Støt Soldater & Pårørende (SSOP) [14]. The study aimed to explore the experiences of children and young people from military families and assess the effect of a five-day residential camp as a psychosocial intervention on their emotional well-being, decision making, confidence, resilience and self-esteem [15].

Ten young people, aged 12–19 (mean = 15.00, SD = 2.54), were recruited using a purposive recruitment from a five-day residential camp in Denmark organized by Støt Soldater & Pårørende (SSOP) for children of military veterans. Six participants were female, and four were male. All had at least one parent who was a veteran with a self-reported diagnosis of Post Traumatic Stress Disorder (PTSD) at the time of the camp. A Danish military veteran is defined as someone who has been deployed in international operations at least once, based on a decision made by the Danish Government, Folketinget, or a minister [16, p.1]. Therefore, the participants’ parents included both active military personnel and veterans who had left the service.

### Method

Secondary data analysis was conducted, based on a previous study exploring the wellbeing of military children attending an activity camp in Denmark [14,16]. The aim of this paper is to use the transcripts of the original study to identify the positive and challenging experiences of children and young people growing up in a military family with parental combat related Post Traumatic Stress Disorder (PTSD)To identify the positive and challenges within the narratives of the participants we are using the ACEs and BCEs as the foundational framework to explore the impact life experiences has on their lives.

### Ethics

As documented in our previous publication [14] ethical approval was obtained through Northumbria Universities ethics committee approval number 17425. Recruitment of young people began on 1st August 2019 and was completed on the 6^th^ August 2019. Due to the participants being under 18 written consent was obtained from their parent/guardian. Additionally, in line with best practice both written, and ongoing verbal consent was acquired from the young person who participated. Any participant who required support during the initial research was supported by the staff at the summer camp, in line with the safeguarding policy and procedures.

### Analysis

The following section provides the details of the screening tools used to analyse the narrative data.

#### Adverse childhood experiences.

The ACEs scale developed by the Kaiser - CDC [3] was used to assess the ACEs of the participants. The scale below table (Table 1) consists of 3 subscale and 10 items, including abuse, neglect, and family dysfunction. Each Yes response is scored as a 1 and a No response scored as 0. The total scores were calculated by the sum of 10 items, with the higher scores indicating greater exposure to adverse events.

**Table 1:** ACEs (Felitti, Schestatsky, Knijnik et al, 1998).

Prior to your 18th birthday:	
1	Did a parent or other adult in the household often or very often… Swear at you, insult you, put you down, or humiliate you? or Act in a way that made you afraid that you might be physically hurt?
2	Did a parent or other adult in the household often or very often… Push, grab, slap, or throw something at you? or ever hit you so hard that you had marks or were injured?
3	Did an adult or person at least 5 years older than you ever… Touch or fondle you or have you touch their body in a sexual way? or attempt or actually have oral, anal, or vaginal intercourse with you?
4	Did you often or very often feel that … No one in your family loved you or thought you were important or special? or Your family didn’t look out for each other, feel close to each other, or support each other?
5	Did you often or very often feel that … You didn’t have enough to eat, had to wear dirty clothes, and had no one to protect you? or Your parents were too drunk or high to take care of you or take you to the doctor if you needed it?
6	Were your parents ever separated or divorced?
7	Was your mother or stepmother: Often or very often pushed, grabbed, slapped, or had something thrown at her? or sometimes, often, or very often kicked, bitten, hit with a fist, or hit with something hard? or ever repeatedly hit over at least a few minutes or threatened with a gun or knife?
8	Did you live with anyone who was a problem drinker or alcoholic, or who used street drugs?
9	Was a household member depressed or mentally ill, or did a household member attempt suicide?
10	Did a household member go to prison?

#### Benevolent childhood experiences.

The Benevolent Childhood Experience Scale (BCEs) was developed building upon existing measures and studies related to positive childhood experiences [17]. Its goal was to fill the gaps in evaluating BCEs that were not sufficiently addressed by other assessment tools. Its creator Narayan, Rivera, Bernstein et al., [17] noted that many of the available tools for measuring positive life experiences often included elements that are not culturally universal, may reflect particular cultural parenting practices, or may mix positive childhood experiences with socioeconomic factors. The BCEs was specifically designed to be a culturally adaptable, ensuring it could be used in all cultural contexts, be that rural, urban, and across all stages of child development, and against all socioeconomic backgrounds [18,17].

As described by Narayan, Rivera, Bernstein et al., [17] in [18] the BCEs scale (Table 2) assesses the presence of 10 positive childhood experiences, using a Yes/No response format, where a Yes is scored as a 1 and a No is scored as 0. The authors [17] grouped positive childhood experiences into three categories, perceived relational and internal security (e.g., there was at least one safe caregiver and beliefs that provided comfort), positive and predictable life quality (e.g., regular meals and a bedtime), and interpersonal support (e.g., a caring teacher). BCEs generate a total score out of 10, with the higher scores indicating more positive childhood experiences. As with the ACEs screening tool, the BCEs screening tool follows a cumulative approach, suggesting that the accumulation of positive events may have more substantial impact on health outcomes than isolated events [19].

**Table 2:** BCEs (Narayan, Rivera, Bernstein et al., 2018).

Prior to your 18th birthday:	
1	Did you have at least one caregiver with whom you felt safe?
2	Did you have at least one good friend?
3	Did you have beliefs that gave you comfort?
4	Did you like school?
5	Did you have at least one teacher who cared about you?
6	Did you have good neighbours?
7	Was there an adult (not a parent/caregiver or the person from #1) who could provide you with support or advice?
8	Did you have opportunities to have a good time?
9	Did you like yourself or feel comfortable with yourself?
10	Did you have a predictable home routine, like regular meals and a regular bedtime?

Through the use of content analysis, the transcribed interview data was re-analysed by the authors of this paper using both the ten-item Adverse Childhood Experiences (ACEs) questionnaire [3] and the ten-item Benevolent Childhood Experience (BCEs) questionnaire [18] to identify ACEs and BCEs within the participants’ narratives. As suggested by Spratt, McGibbon & Davidson [20] using the ACEs screening tool has its advantages of being used both in large population surveys and specific population studies, which as he describes, provides data which offers sufficient weight to establish probability for a range of outcomes (including health and social circumstances).

McGavock and Spratt [21] acknowledge that the ACE screening tool was initially designed to be used as an interviewer or self-reporting tool. However, they argue it has its uses as a tool for qualitative researchers for secondary data analysis, as it provides a systematic means of analysing data, thus, bringing light to previously hidden patterns or text, which in turn, could provide a bridge to link narratives to outcomes. The major disadvantages of using the ACE screening tool as a method of qualitative data analysis is that you will only ever get partial scores, as we have only screened the content provided, and not been able to ask the full set of screening questions, thus reducing its accuracy [20].

That said, as a systematic tool to identify ACEs within qualitative data we thought it would be useful to test on our data set. Building on McGavock and Spratt’s [21] use of the ACEs screening tool within qualitative analysis it was felt the BCEs screening tool would also be an appropriate tool to identify benevolent childhood experiences within the same data set, to understand both adversity and benevolence.

## Results

In the following tables, the numbers in the top row represent the 10 ACEs and BCEs questions, with the participant numbers running down the first column. Tables 3 and 4 represent the ‘raw’ data form, due to participant allocation of number. In Tables 5 and 6, the tables demonstrate the descending numerical number of ACEs and BCEs of each participant.

**Table 3:** (Key ‘X’ is yes).

ACE	1	2	3	4	5	6	7	8	9	10
A14	X	X		X		X			X	
A17	X			X		X			X	
A25						X				
A28		X				X			X	
A33				X					X	
A38						X			X	
A43	X					X			X	
A46				X		X			X	
A47									X	
A48	X					X			X	

**Table 4:** (Key ‘X’ is yes).

BCE	1	2	3	4	5	6	7	8	9	10
A14	X	X	X			X	X	X		X
A17	X	X	X	X	X	X	X	X	X	X
A25	X	X				X	X	X		X
A28	X	X		X	X	X	X	X		X
A33	X					X	X	X		X
A38	X	X		X	X		X	X		X
A43	X	X	X			X	X	X	X	X
A46	X	X	X	X	X		X	X	X	X
A47	X	X	X		X	X	X	X	X	X
A48	X	X		X	X		X	X		X

**Table 5:** (Key ‘X’ is yes).

ACE	1	2	3	4	5	6	7	8	9	10
A14	X	X		X		X			X	
A17	X			X		X			X	
A43	X					X			X	
A48	X					X			X	
A46				X		X			X	
A28		X				X			X	
A38						X			X	
A33				X					X	
A47									X	
A25						X				

**Table 6:** (Key ‘X’ is yes).

BCE	1	2	3	4	5	6	7	8	9	10
A17	X	X	X	X	X	X	X	X	X	X
A46	X	X	X	X	X		X	X	X	X
A47	X	X	X		X	X	X	X	X	X
A28	X	X		X	X	X	X	X		X
A43	X	X	X			X	X	X	X	X
A38	X	X		X	X		X	X		X
A48	X	X		X	X		X	X		X
A14	X	X	X			X	X	X		X
A25	X	X				X	X	X		X
A33	X					X	X	X		X

The majority of the participants (nine) had a parent with mental ill health. As the initial study highlighted and one of the reasons the participants attended SSOP were for support due to parental combat related PTSD. Due to the presence of parental poor mental health, these participants all had one ACE score. By contrast one participant did not have a parent with PTSD but attended due to being a step sibling to another participant. The group (9) with a parent with PTSD has a mean ACE score of 2.7. The reordering in Table 5 demonstrates that the group (9) with a parent with PTSD has additional ACEs, with only one of the group’s (9) not having an additional ACE. Of note, the independent emotional health and well-being measures were used during the larger project and the result can be found in Watson & Osborne, [16].

On the contrary as shown in Table 6, one participant had 10 BCEs, two had 9 BCEs and the remainder recording 8 or lower BCEs. Interestingly, all the participants reported having BCEs 1, 7, 8 and 10 (mean 4). Meaning they all had a caregiver who they felt safe with, had an external adult non-caregiver who they could seek support or advice from, had opportunities to having a good time and had a predicable home routine.

### Summary of the results

In summary, the above findings demonstrate whilst the ACEs tool assess negative experiences, the BCEs tool assess the positives or supportive aspects of the participants life. It is these positives which can in some circumstances negate the negatives. This comparison in the screening tools is critical to the aim of the paper, which seeks to understand both adverse and positive childhood experiences influence the health and well-being of the participants.

The findings of this secondary analysis add to the literature regarding being a MCCYP and living with a parent with combat related PTSD, and the additional adversities they may face. Whilst research in this area is limited, there has been acknowledgment that being a MCCYP does have its challenges with regards to parental deployment, re-integration, transition and poor parental mental health issues, this analysis offers insight into future issues MCCYP may face with regards their own health and well-being due to their ACE score. Besides the predominant factor of the participants having a parent with PTSD (see Table 3), the addition of parental separation (eight participants), having a caregiver swear, insult, or make them feel afraid of being hurt (4) and have never felt loved, important or the family not looking out for each other contributes to the ACE score of the child (4). There are documented relationships regarding parental combat related PTSD and its effects on children and young people within the home [22–24]. Therefore, we might speculate that the additional ACEs besides parental PTSD, family separation, feeling afraid and not feeling loved are a byproduct of the symptoms of living with parental combat related PTSD, and thus the difficulties with emotional engagement and meeting the child’s emotional needs [14].

The findings within the BCEs narrative screening offers a different side to the coin, in that all of the participants had four benevolent experiences. Importantly, all the participants had a caregiver who they felt safe with. This finding supports Chandra, Lara-Cinisomo, Jaycox et al., [25] who found the positive emotional health of the MCCYP during parental deployment and reintegration/transition is reliant on the nondeployed parents own positive mental health. Having a parent with positive mental health was understood by the participants as the emotional stability within the family home which also provided the participants with a sense of routine and structure, enabling them to have a secure base and potentially support their abilities to thrive in what can be at times a hostile home environment due to parental combat related PTSD [26–28].

### The role of practitioners in letting stories breathe

#### Identify, Connect & Engage (ICE).

Given the prevalence of ACEs within the population of children and young people, an increasing number of health and social care providers, schools and services within the charity sector have invested in the use of trauma informed care. Trauma Informed Care (TIC) is characterised by child or young person-centred, which may also include family centred practices needed to facilitate trust, safety, respect, collaboration and importantly shared power. It is well documented that health and social care professionals, alongside the teaching workforce are in a privileged position, due to their access, to support MMCYP who may have a number of ACEs. It is this access which can provide early identification and connection with MCCYP as well as their families to positively engage with appropriate intervention to address potential trauma by building on the child or young person’s BCEs, which have been described to counter ACEs [28].

**Fig 1:**
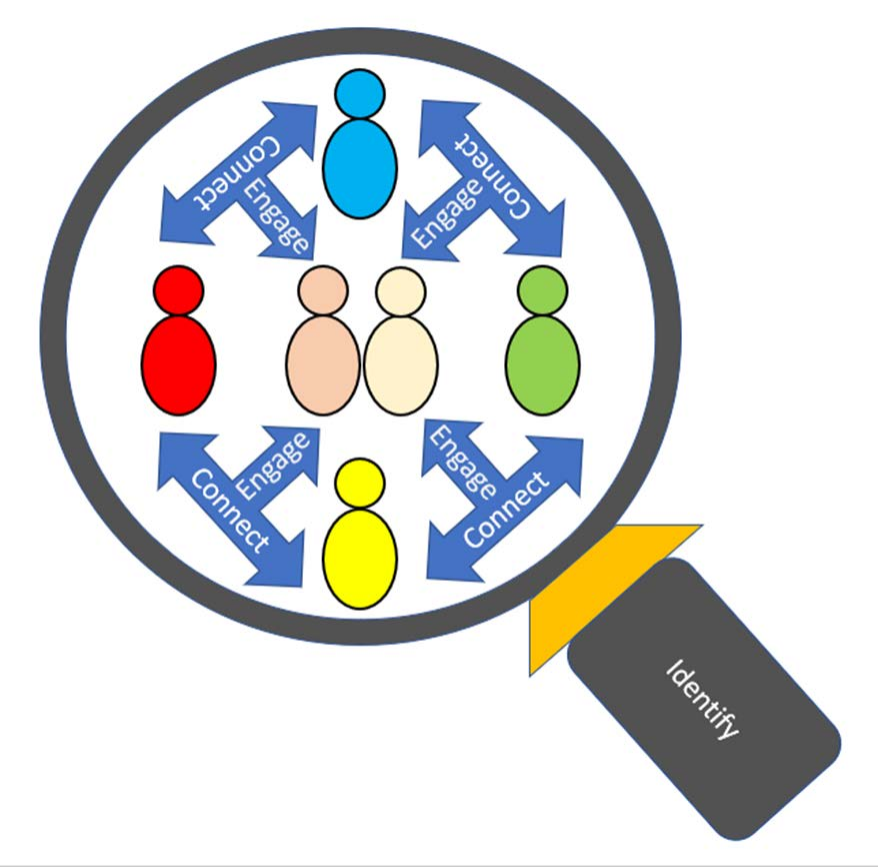
Identify, Connect, Engage (ICE) Model Watson and Farrell, 2023. Visual representation of the three-phase framework for holistic, collaborative engagement.

The Identify, Connect, and Engage (ICE) model (Fig 1), developed by Watson & Farrell [29], promotes a holistic, collaborative approach to addressing an issue or engaging with an individual. This model focuses on identifying the issue or person, establishing a connection, and ensuring active involvement. The following section will apply a public health approach to trauma-informed principles and practices, using the ICE model as its framework.

#### Identify.

Public health focuses on improving the overall health of a population by improving the health of individuals within that population using various means, including disease prevention, and monitoring and modifying environmental factors alongside health screening [30]. Importantly, whilst the lens of this paper sits under the umbrella of public health, the use of ICE is transferable to all agencies working with YP (everybody’s business not just the public health workforce). Therefore, identifying, public health approaches to understand and address adversity within MCCYP involve interventions delivered at a population level by all involved. Along with targeting resources effectively through increased understanding of a population, identifying the root causes and looking behind a presenting illness or health issues. As previously discussed, the interest in detecting or identifying ACEs has rapidly developed as a public health concern due to the distinctive links between ACEs and many non-communicable diseases [31]. It can therefore be argued that early identification of adversities has the potential to act as an appropriate method in the creation of a formulation for intervention, which in turn can go some way to mitigate the negative impact of childhood adversity [32,33] and promote better health and well-being outcomes for MCCYP.

It is well documented that MCCYP are a hidden population of children and young people [34–38] and due to the secrecy surrounding military life, are often seldom heard and seldom asked about their lived experiences. Professionals who actively and knowingly work with MCCYP including, children’s nurses, school nurses, health visitors, social workers and teachers encounter MCCYP who have a history of trauma [39]. There are some significant positives which professionals can adopt when they have identified the needs of a MCCYP, by understanding and inquiring into the narratives of MCCYP. Inquiry based practices enable professionals to identify potential ACEs within their assessment processes, without actually relying on the tick box exercise of an ACEs screening tool. Increasingly across the education, and health and social care sector there is recognition that those who come in contact with children and young people are trained to treat everyone with compassion, care and consideration, as they are yet to hear and understand the untold narratives of those they are caring for. Importantly, professionals need not always seek to investigate by screening for ACEs, which may cause or trigger a trauma response, but should recognise that a MCCYP behaviour or actions may provide an insight into the potential of hidden trauma. It is these behavioural presentations which require positive and purposeful connection.

#### Connect.

Through meaningful and compassionate connection, TIC providers may be able to gain the trust needed to build relationships with MCCYP that provide the insights into their adverse and positive experiences. Social connectedness plays an important role in creating a supportive network and environment that can foster resilience [14,40], yet MCCYP and families may face barriers such as stigma related to feeling judged by social support services [41], geographic decreases in access, and lack of confidence to seek services [42]. Civilian social support providers may not be able to easily identify MCCYP or be equipped with the knowledge on the unique experiences that MCCYP face [43] and therefore, feel they cannot adequately understand the challenges they are facing. Training and education on these experiences are essential to facilitating connections in which MCCYP feel understood and accepted. TIC should take steps towards providing mental health, military awareness, and mentoring training for social support personnel to develop this [29]. TIC should also aim to foster open communication through educational and social programmes between groups of peers or personnel to allow for positive connections [41].

Fostering connection goes beyond simply identifying experiences and marking them within a predetermined selection. Building positive relationships over time can allow social support services to actively listen to the narratives of MCCYP and validate the emotions arising. Uncovering experiences through untold stories rather than a screening tool can build rapport and make for a mutually enriching connection in a trauma informed and experience sensitive manner. Building these meaningful connections that are specific to the needs and experiences of MCCYP serve as positive childhood experiences that have influences on future trajectories. It may be beneficial for TIC to encourage connection on different levels, including self, family, and community, due to the various benefits that understanding their experiences, gaining support, and reintegrating within the community can grant MCCYP. Encouraging connection to self, family, and friends can create a great stepping stone towards engaging with communities and enabling communication and action.

#### Engage.

**Fig 2:**
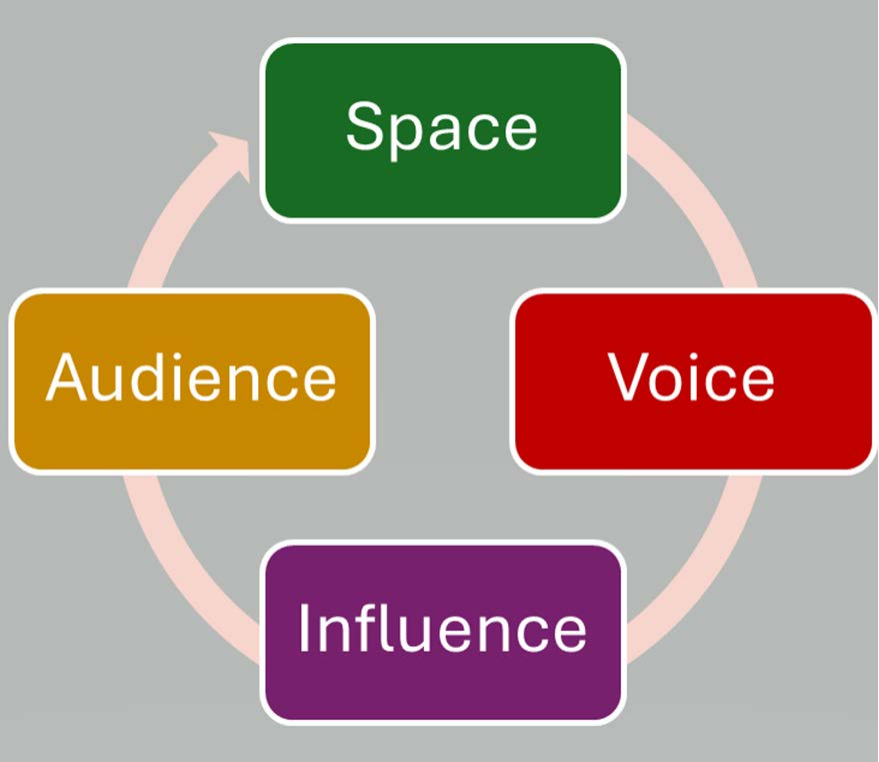
The Lundy Model Lundy (2007). Visual representation of the four interconnected elements: Space, Voice, Audience, and Influence.

The Lundy Model [43] enables practitioners to engage in enabling children’s participation in assessment, formulation and practice. The model is built on four key elements—Space, Voice, Audience, and Influence (See Fig 2) which work together to create a dynamic process in which participants’ voices are heard in specific contexts (spaces), impacting the decision-making process and influencing those who receive (audience) the information. Within the original study the concepts of space, voice, audience and influence were applied to understand how participants contributed to sharing their lived experiences of being a child or young person with a parent with PTSD.

Using the Lundy model ensures practitioners positively engage with MCCYP and actively listen to their stories and are actively heard and acted upon in healthcare and social care settings. In this context, professionals can adopt the Lundy Model to guide their engagement with MCCYP and assess their needs, providing an inclusive and child-centred approach to care.

For MCCYP, ***space*** means creating a safe, supportive environment where children can freely express their feelings about the stressors they face, such as parental deployment or mental health challenges within the family. Practitioners can facilitate this through age-appropriate communication techniques, building rapport and trust [44–46]. The ***voice*** element is then realised when practitioners actively engage children, encouraging them to share their experiences through direct conversations and allow their narratives to breathe [47,48]. Importantly, practitioners provide the ***audience*** by listening attentively to the MCCYP’s concerns and ensuring that both the child’s and their families input are considered. Lastly, ***influence*** is a critical aspect, as practitioners, in collaboration with MCCYP, their families and interdisciplinary teams, ensure that the child’s expressed needs and concerns lead to concrete actions, ensuring that their experiences, challenges, and voices are meaningfully co-produced and integrated into the care process [49,50].

Importantly, the findings from this study illustrate the many health and social supports MCCYP receive. Prior research has shown that the lower rates of poverty and higher parental resources of MCCYP [51], coupled with MCCYP residing in households with higher education levels and higher rates of two parents, contribute to greater social supports than non-MCCYP families. MCCYP also reside in pre-existing social networks, due to the military, which may enhance their ability to have connected mentors outside of their household to foster resilience and BCEs [52]. A large proportion of research on MCCYP focuses on the negative experience MCCYP may confront with caregivers who may be away for extended periods of time, and caregivers dealing with the effects of trauma. While ACEs among MCCYP are important to study, there have been only a few studies that look at positive childhood experiences [7]. Our research from this study confirms this work and shows that MCCYP may also have nurturing and supportive environments to foster BCEs and resiliency.

In summary, the ICE model provides a robust framework for identifying, connecting with, and engaging MCCYP in ways that acknowledge their unique experiences and foster resilience. By adopting trauma-informed practices and prioritising the building of trust and meaningful connections, professionals can help mitigate the negative impacts of ACEs and promote the health and well-being of MCCYP.

## Discussion

The findings from this study align with the aim of ‘letting stories breathe’ by using the ACEs and BCEs screening tools when analysing the narratives of MCCYP be that in real time or retrospectively. The ACEs tool highlights the negative experiences these MCCYP face, such as parental separation, parental mental health issues, substance use and emotional neglect. However, the BCEs tool reveals how positive supportive aspects of their lives, for example having a caregiver they feel safe with, have friends they can speak to, opportunities to have a good time can potentially counter the adversities. This balance between ACEs and BCEs is essential in providing a holistic understanding of MCCYP emotional health and well-being [12,18,17].

The findings of the secondary analysis add to existing literature on the challenges faced by MCCYP, particularly those living with a parent who has combat-related PTSD [1,2,7,14]. While ACEs such as parental poor mental health, separation and substance use are well documented, this study uncovers additional adversities within this population of MCCYP, for example emotional neglect and a lack of feeling loved, which contributed to the ACE score. These findings suggest that the negative experiences these MCCYP face may be a direct result of the emotional disengagement linked to living with a parent with combat related PTSD [18,34].

However, in contrast, the BCEs findings offer a different perspective, demonstrating the strength of these MCCYP. Despite the identified ACEs within their stories, all participants reported at least four positive experiences, in particular having a caregiver they feel safe with. This finding supports previous research conducted by Chandra et al., [25], which highlighted that the emotional health and well-being of the stay-at-home or non- PTSD parent is crucial to the emotional health of the child or young person. The presence of positive caregiving, structure, and stability provides these MCCYP a secure base [53,54], which has the potential to mitigate or counter the negative effects of parental combat related-PTSD. These findings emphasize the importance of BCEs in fostering the potential of positive outcomes for this population who have a number of ACEs.

The sole use of TIC may limit the effectiveness of addressing the diverse needs of MCCYP, by focusing primarily on trauma, or adversities, without considering the individual nuances of each MCCYP experience. Whilst TIC is essential in recognising the impact of trauma like parental combat related PTSD, neglect or emotional abuse, it may overlook the important factors such as protective relationships and thriving outside of the family home. Therefore, a more tailored and specific approach is needed that incorporates both the trauma or adversities experienced and the unique strengths or support each MCCYP experiences. By combining a holistic approach to TIC will ensure a more comprehensive understanding. The ICE Model [29] promotes a holistic, collaborative approach to addressing and issue or engaging with an individual. This model provides researchers and practitioners with a conceptual framework which focuses on identifying the issue or person, establishing a connection, and ensuring positive and active engagement. In the analysis of secondary data, the ICE model was used to demonstrate how researchers, clinicians and front facing service providers could identify both the positive and challenges of MCCYP lives, connect with their experiences and actively engage in their stories when conducting assessments or conducting retrospective narrative analysis [33,44,46]. Moreover, the inclusion of The Lundy Model [43] offers an epistemological bricolage for layering additional theory to the ICE Model [29]. The Lundy Model provides an additional framework for enabling the voice of MCCYP during the process of research and clinical practice. The four elements of the Lundy Model work together to create a dynamic process in which participants are provided a safe space to their stories breath [55]. The use of the Lundy Model not only ensures that the voice of MCCYP are heard but also fosters an environment where MCCYP feel empowered to express themselves fully, knowing their perspectives are valued and can influence their own futures [13,19,32].

## Limitations

This secondary data analysis has several limitations that impact its generalisability, particularly in its use of the ACE and BCE narrative screening tools. The small sample size of 10 participants is insufficient for robust quantitative analysis and limits the ability to validate subgroups or detect subtle differences related to demographics. The ACE questionnaire, used as a secondary tool, provides incomplete data and likely underestimates the mean scores of adverse experiences. Similarly, while the BCE tool highlights positive experiences, its reliance on subjective self-reports and the small sample size reduces its generalisability. The overlap of these tools underscores the need for a more integrated approach to assess both adversity and protective factors comprehensively.

Despite these limitations, the findings highlight important trends. MCCYP caring for parents with mental illness face significant adversities. However, the BCE results emphasise the protective value of a safe and supportive caregiver, aligning with prior research that links having a positive adult within a child or young person’s life improved emotional outcomes for MCCYP. This stability can help MCCYP develop resilience, even in challenging home environments such as those affected by parental combat-related PTSD. Future large-scale studies should combine ACE and BCE tools to explore the interaction between adversity and protective factors, providing deeper insights into how to support MCCYP effectively. Further work is needed to ensure that trauma informed principles and practices are not only providing supportive environments, but also actively seeking to validate and engage MCCYP and their families in appropriate interventions. Uncovering stories and narratives requires engagement that does not give a fixed meaning to their experiences and allows for a deeper dive into their reflections and contexts to enable practitioners to find meaning within the told story. Engaging and actively seeking the implementation of support, relationships and awareness between MCCYP and practitioners will allow stories to provide a richer understanding of the unique adverse and positive childhood experiences that impact their long-term outcomes.

## Conclusion

As we continue to develop our understanding and awareness of the impact and prevalence of ACEs and BCEs in the MCCYP population, it is imperative that we also focus on uncovering these experiences and building on the support mechanisms and strategies for MCCYP. The impact of exposure to ACEs on physical and emotional well-being has been pivotal in shaping the development of trauma-informed policies and programmes. Moreover, it has prompted discussion for changes in practice, with a focus on facilitating early identification to help mitigate the long-term effects of adversity in childhood that would therefore enable policy makers and service providers to improve the positive aspects of growing up in a military family. These efforts are crucial in addressing and mitigating the likelihood of physical, emotional, and social morbidities associated with childhood trauma, but also identify the positives of military life and how MCCYP develop resilience and ways to develop positively. By actively seeking out and implementing effective support systems and strategies and actively inquiring into the narratives and stories from MCCYP we can work towards promoting a more rounded picture of military life through the eye of those who experience it.

## Supporting information

S1 Checklist Inclusivity in global research. (DOCX)
